# Mitochondrial morphology and energy metabolism in reprogrammed porcine expanded potential stem cells

**DOI:** 10.5713/ab.24.0521

**Published:** 2024-10-24

**Authors:** Yun Ju Lee, Jae Hoon Song, Je Woo Lee, Tae Kyung Hong, Sang Jun Uhm, Kwonho Hong, Jeong Tae Do

**Affiliations:** 1 Department of Stem Cell and Regenerative Biotechnology, Konkuk Institute of Technology, Konkuk University, Seoul 05029, Korea; 2 Biotechnology Research Institute, MGENSolutions Co., Ltd., Seoul 06591, Korea; 3 Department of Animal Science, Sangji University, Wonju 26339, Korea

**Keywords:** Cellular Metabolism, Cellular Reprogramming, Extended Pluripotent Stem Cells (EPSCs), Mitochondrial Dynamics, Pig Cells

## Abstract

**Objective:**

Expanded potential stem cells (EPSCs) are stem cells that can differentiate into embryonic and extraembryonic lineages, including extraembryonic endoderm and trophoblast lineages. Therefore, EPSCs have great potential in advancing regenerative medicine, elucidating disease mechanisms, and exploring early embryonic development. However, the generation and characterization of EPSCs in pigs have not been thoroughly explored. In this study, we successfully generated porcine EPSCs (pEPSCs).

**Methods:**

We reprogrammed porcine fetal fibroblasts (PFFs) using an integration-free method with Sendai virus vectors.

**Results:**

The resulting pEPSCs expressed key pluripotency markers and demonstrated the ability to differentiate between embryonic and extraembryonic lineages. Notably, reprogramming into pEPSCs was associated with a transformation of mitochondrial morphology from the elongated form observed in PFFs to a globular shape, reflecting potential alterations in energy metabolism. We observed significant remodeling of mitochondrial morphology and a subsequent shift towards glycolytic energy dependence during the reprogramming of PFFs into pEPSCs.

**Conclusion:**

Our findings provide valuable insights into the characteristics of EPSCs in pigs and highlight their potential applications in regenerative medicine, disease modeling, and emerging fields such as cell-based meat production.

## INTRODUCTION

Pluripotent stem cells (PSCs) can self-renew *in vitro* and differentiate into all three germ cell layers *in vivo* and *in vitro* [1,2]. Naïve PSCs exhibit an unbiased potential to differentiate into all three germ layers and form chimeras after blastocyst injection, whereas primed PSCs show limited differentiation potential, particularly *in vivo* [3]. However, both naïve and primed PSCs showed limited contribution to extraembryonic lineage tissues [4].

Yang et al [5] introduced expanded potential stem cells (EPSCs), which can differentiate into both embryonic and extraembryonic lineages. This potential has been demonstrated in mice, humans, pigs and bovine embryos [5–8]. Due to their ability to differentiate into extraembryonic lineages, EPSCs are sometimes considered totipotent-like stem cells. Unlike naïve PSCs derived from the inner cell mass (ICM) of blastocysts, EPSCs are derived from 8-cell stage embryos where lineage specification has not yet occurred.

PSCs can also be generated by reprogramming somatic cells using specific reprogramming factors, creating induced PSCs (iPSC) [9]. Integration-free methods, such as Sendai-viral vectors, plasmid vectors, recombinant proteins, synthetic modified RNAs, micro RNAs and small molecules, have been developed to avoid the instability caused by integration-inducing methods [10–15]. Among these, Sendai virus vectors are particularly effective due to their high reprogramming efficiency and non-integrative nature [11].

Energy metabolism is essential for cell survival, with different cell types employing distinct metabolic pathways [16]. Mitochondrial oxidative phosphorylation (OXPHOS) is an efficient cellular process that produces adenosine-5′-triphosphate (ATP) by utilizing oxygen [17]. Differentiated cells with mature mitochondria generally employ OXPHOS for energy production [18]. In contrast, actively proliferating cells such as stem cells and cancer cells predominantly rely on aerobic glycolysis for energy production. Therefore, the mitochondrial morphology and energy metabolism undergo dynamic changes during the reprogramming of differentiated cells into iPSCs, characterized by a metabolic shift from OXPHOS to glycolysis [19,20]. Along with metabolic changes, the elongated mitochondria in fibroblasts shift to a globular shape during reprogramming into a pluripotent state [20]. However, characterization of mitochondrial function and energy metabolism in porcine iPSCs or EPSCs remains limited.

In this study, we aimed to establish porcine EPSCs (pEPSCs) from porcine fetal fibroblasts (PFFs) using an integration-free Sendai virus vector. The established pEPSCs showed the potential to differentiate into both embryonic and extra-embryonic lineages, confirming their expanded differentiation potential. We further investigated the distinctive features of mitochondrial morphology, oxygen consumption rate (OCR), and extracellular acidification rate (ECAR) in pEPSCs to understand their energy metabolism.

## MATERIALS AND METHODS

### Derivation and culture of porcine fetal fibroblast

PFFs were obtained by surgically opening the uterus of a pregnant female domestic pig at embryonic day 35 (E35) for fetal recovery. The heads, limbs, and internal organs of the fetuses were removed, and the remaining tissues were digested for 30 min with 0.25% trypsin-EDTA (25200-072; Gibco, Waltham, MA, USA). The digested tissues were plated in a 100 mm culture dish. PFFs were maintained in Dulbecco’s modified eagle medium (DMEM) low glucose medium supplemented with 10% fetal bovine serum (FBS) and 1x Penicillin-Streptomycin ([P/S] 15140-122; Gibco) under 5% CO_2_ at 37°C. All animal studies were reviewed and approved by Institute of Animal Care and Use Committee (IACUC) of Konkuk University (#KU23148).

### Reprogramming porcine fetal fibroblasts by Sendai-virus

**Figure 1:**
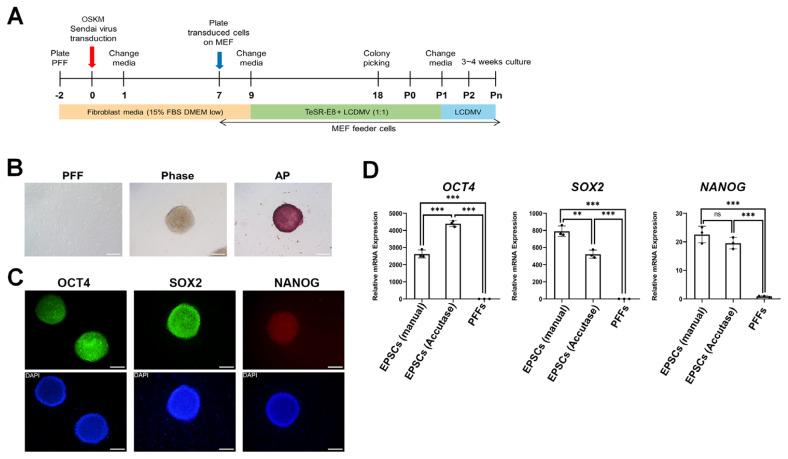
Generation and characterization of porcine extended pluripotent stem cells. (A) Schematic illustration for the establishment of pEPSC using Sendai virus transduction (OSMK) and LCDMV culture media. (B) Morphology of PFF cells before transduction (left), at passage 9 after the establishment of pEPSCs (middle), and a representative image of AP-staining (right). Scale bars: 100 μm. (C) Immunocytochemistry analysis of pEPSCs for Oct4, Sox2, Nanog (Pluripotent markers). Scale bars: 200 μm. (D) RT-qPCR analysis of pluripotent markers (Oct4, Sox2, Nanog) in pEPSCs with different sub-culture methods compared to PFFs. pGapdh was used as a housekeeping gene for sample normalization. PFFs, porcine fetal fibroblasts; MEFs, mouse embryonic fibroblasts; FBS, fetal bovine serum; DMEM, Dulbecco’s modified eagle medium; pEPSCs, porcine expanded potential stem cells; AP, alkaline phosphatase; EPSCs, expanded potential stem cells. Student’s t-test: ns = 0.208, ** p = 0.004, *** p<0.001.

The scheme for reprogramming PFFs into iPSCs is shown in Figure 1A. Two days after seeding fibroblast, 5×10^4^ live cells were transduced with Sendai virus vectors encoding *OCT4*, *SOX2*, *KLF4* (*OSK*), and *C-MYC* (*M*) (CytoTune-iPSC2.0 Sendai Reprogramming Kit, A16517; Life Technologies, Carlsbad, CA, USA) at a multiplicity of infection. The following day, the medium was replaced with fresh fibroblast medium and refreshed every other day for six days. On day 7 post-transduction, 5×10^4^ cells/mL fibroblasts were plated on 7.5×10^4^ cells/mL CF1 feeder culture dishes and replaced with TeSR-E8 medium and hLIF, CHIR99021, DiM, MiH, vitamin C (LCDMV) medium (1:1) after 48 h. Colonies were observed until day 18 and transferred to CF1 seeded 4-well dishes (p0). Subculturing was performed manually using Pasteur pipettes in LCDMV media.

### LCDMV cell culture system

The pEPSCs were cultured in LCDMV culture media following established protocols [7]. pEPSCs from colony picking were attached to mitotically inactivated CF1 mouse embryonic fibroblasts (MEFs) and maintained in LCDMV medium. The LCDMV medium consisted of 50% Neurobasal (21103-049; Gibco), 50% DMEM/F12 (11320-033; Gibco), 5% Knockout serum replacement (10828-028; Gibco), 1×N2 (17052-048; Gibco), 0.5×B27 (17504-044; Gibco), 10 ng/mL human recombinant leukemia inhibitory factor (LIF1010; Millipore, Burlington, MA, USA), 1 μM CHIR99021(C-6556; LC laboratories, Woburn, MA, USA), 2 μM S-(+)-dimethindene maleate (Tocris, 1425), 2 μM minocycline hydrochloride (sc-203339; Santa Cruz, Dallas, TX, USA), and 40 μM 2-Phospho-L-ascorbic acid trisodium salt (49752; Sigma, St. Louis, MO, USA). pEPSCs were passaged every 2 to 3 days onto freshly inactivated CF1 MEF, and the medium was changed daily.

### Alkaline phosphatase staining

Cells were washed with phosphate-buffered saline (PBS) and fixed with 4% paraformaldehyde (PFA) for 5 min at room temperature. Alkaline phosphatase (AP) staining was performed using an AP kit II (00-0055; Stemgent, Beltsville, MD, USA) according to the manufacturer’s protocol.

### Embryoid body formation and *in-vitro* three-germ layer differentiation

Cells were dissociated into single cells and suspended in differentiation medium to form embryoid bodys (EBs) by suspension culture in a 60 mm petri dish. The differentiation medium consisted of DMEM low glucose supplemented with 15% FBS, 1×penicillin-streptomycin-glutamine ([P/S/G] 10378016; Gibco), 0.1 mM non-essential amino acids, and 1 mM β-mercaptoethanol.

EBs were harvested after 11 days, washed, and attached to a 0.15% porcine gelatin-coated culture dish. Differentiation proceeded for approximately 2 weeks, with medium changes every 2 days.

### Immunocytochemistry

Cells were fixed with 4% PFA for 20 min at 4°C. After washing with PBS, cells were treated with 0.3% Triton X-100 in PBS for 10 min and blocked with PBS containing 3% bovine serum albumin (BSAS0.1; Bovogen, Keilor East, Australia) for 1 h at 25°C. Primary antibodies were applied at the following concentrations: OCT4 (1:500, rabbit), NANOG (1:500, rabbit), SOX2 (1:500, rabbit), tubulin beta III isoform (TUJ1; 1:500, mouse), BRACHYURY(T; 1:500, mouse), smooth muscle actin (SMA; 1:500, mouse), SOX17 (1:500, goat), GATA4 (1:200, rabbit), CDX2 (1:1250, rabbit), EOMES (1:500, rabbit), GATA3 (1:500, mouse). The following day, the primary antibodies were removed, and cells were washed thrice with PBS for 10 min. After washing, cells were labeled with fluorescence-conjugated secondary antibodies to detect the primary antibodies at the following concentrations: Alexa Fluor 488 (1:500) and Alexa Fluor 568 (1:500), Alexa Fluor 647 (1:500) for 2 h at 25°C. Lastly, they were treated with 4′,6-diamidino-2-phenylindole (DAPI) or Hoechst in 0.3% Triton X-100 in PBS for 3 min at 25°C and washed. The antibodies used for immunocytochemistry are listed in Table 1.

### RNA isolation and real-time reverse transcription quantitative polymerase chain reaction

Total RNA was extracted using TRIzol reagent, and RNA concentration was measured using a Nanodrop (Thermo Scientific, Waltham, MA, USA). cDNA was synthesized from 1 μg total RNA using SuperScriptTM III Reverse Transcriptase (18080-044; Invitrogen, Waltham, MA, USA), Oligo(dT)12-18 Primer (18418-012; Invitrogen), and 10 mM dNTP Mix (18427-013; Invitrogen). Real-time polymerase chain reaction (PCR) was performed using TOPrealTM qPCR 2X PreMIX (RT500M; Enzynomics, Daejeon, Korea) and results were analyzed using a Roche LightCycler 5480 (Roche, Basel, Switzerland). Primers used for quantitative reverse transcription (qRT)-PCR are listed in Table 2.

### Electron microscopy

For transmission electron microscopic (TEM), cells were fixed in 4% PFA and 2.5% glutaraldehyde in 0.1 M phosphate buffer for 3 h. Samples were post-fixed in 1% osmium tetroxide for 30 min, dehydrated in graded ethanol series (50%, 70%, 80%, 90%, 95%, and 100%), and polymerized in Epon 812 overnight at 60°C. Ultrathin sections were cut to a thickness of approximately 60 to 70 nm using an ultramicrotome (Leica Ultracut UCT, Wetzlar, Germany). Sectioned slices were collected on grids (200 mesh) and stained with 2% uranyl acetate and lead citrate. The prepared grids were examined under a transmission electron microscope (JEM 1010; JEOL, Tokyo, Japan) operating at 60 kV.

### Mitochondrial length measurement

Mitochondrial length and the maximum (Max)/minimum (Min) ratio were analyzed using electron microscopy. Measurements were made using Image J 1.53 software (NIH), and over 25 mitochondria were measured per sample for data analysis [16].

### Oxygen consumption rate analysis and extracellular acidification rate analysis

OCR was measured using a Seahorse XFp analyzer (Seahorse Bioscience, North Billerica, MA, USA). PFFs (7.5×10^4^) and pEPSCs (2×10^5^) were cultured for 16 h after seeding in Matrigel-coated plates (356234; Corning, Corning, NY, USA). The medium was replaced with XFp base medium supplemented with d-glucose (103577-100; Agilent Technologies, Santa Clara, CA, USA), sodium pyruvate (103578-100; Agilent Technologies), and l-glutamine (103579-100; Agilent Technologies). OCR measurements were obtained after injection of oligomycin (1.5 μM), Carbonyl cyanide p-(trifluoromethoxy) phenylhydrazone (FCCP) (0.1 μM), and rotenone/antimycin A (Rot/AA) (0.5 μM) (Agilent Technologies), according to the manufacturer’s instructions. On the other hand, ECAR measurements were taken after injection of Rot/AA (0.5 μM) and 2-DG (80 mM) (Agilent Technologies), according to the manufacturer’s instructions.

### Statistical analysis

All experiments were performed in triplicate. Data are presented as the mean±standard deviation and analyzed using GraphPad Prism 8. Statistical significance was evaluated using Student’s t-test. Kruskal–Wallis test and Dunn’s post hoc test were used to analyze mitochondrial morphology. Statistical significance was set at p<0.05.

## RESULTS

### Generation and characterization of porcine expanded potential stem cells

PFFs obtained at 35 days of pregnancy were reprogrammed using Sendai virus-mediated delivery of *OCT4*, *SOX2*, *KLF4*, and *C-MYC* (*OSKM*) reprogramming factors. By day 7 post-transduction, PFFs were seeded onto feeder-layered plates at a density of 5×10^4^ cells (Figure 1A). Subsequently, on day 9, transduced PFFs were transitioned to a 1:1 mixture of TeSR-E8 and LCDMV media until reaching passage 1 (P1). Approximately 18 days post-transduction, compacted dome-like colonies emerged, which were then subjected to continuous subculturing. Two subculture protocols were tested: manual subculture using Pasteur pipettes and single-cell dissociation using Accutase, with subsequent cultures exclusively using LCDMV medium (Figure 1A).

Reprogrammed pEPSCs exhibited dome-like colony morphology and positive staining for AP (Figure 1B). Immunocytochemical analysis confirmed robust expression of pluripotency markers OCT4, SOX2, and NANOG, although NANOG staining was not strong. Quantitative real-time RT-PCR (RT-qPCR) further validated significantly elevated expression levels of *NANOG* as well as *OCT4* and *SOX2* compared with PFFs across both subculture protocols (manual and Accutase) (Figure 1D), underscoring successful reprogramming using Sendai virus and LCDMV media.

### Expanded differentiation potential of porcine expanded potential stem cells into embryonic and extraembryonic lineages

**Figure 2:**
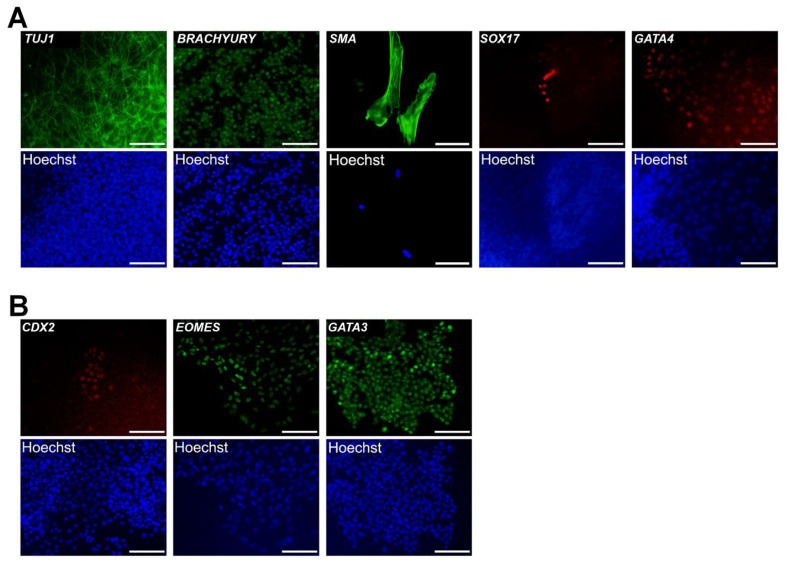
Differentiation of pEPSCs into embryonic and extraembryonic lineages. (A) Immunocytochemistry analysis of the random differentiation of pEPSCs *in vitro* using markers for three germ layers: TUJ1 (ectoderm), Brachyury and SMA (mesoderm), and SOX17 and GATA4 (endoderm). Scale bars: 100 μm. (B) Immunocytochemistry analysis of pEPSC differentiation using extra-embryonic lineage markers: CDX2 and EOMES (trophoblast), and GATA3 (extra embryonic endoderm). pEPSCs, porcine expanded potential stem cells. Scale bars: 100 μm.

One of the distinctive characteristics of EPSCs is their potential to differentiate into embryonic and extra-embryonic lineages [5,21]. To test this extended potency, pEPSCs were differentiated *in vitro* via EB formation. They were able to differentiate into all three germ layers: ectoderm (TUJ1^+^), mesoderm (BRACHYURY^+^, SMA^+^), and endoderm (SOX17^+^, GATA4^+^), as confirmed by immunocytochemical analysis (Figure 2A). Additionally, the ability of pEPSCs to differentiate into extraembryonic lineages was confirmed using trophectoderm markers (CDX2 and EOMES) and a primitive endoderm marker (GATA3) (Figure 2B). Collectively, these results demonstrated that the established pEPSCs are capable of differentiating into both embryonic and extraembryonic lineages.

### Morphological changes of mitochondrial during reprogramming

**Figure 3:**
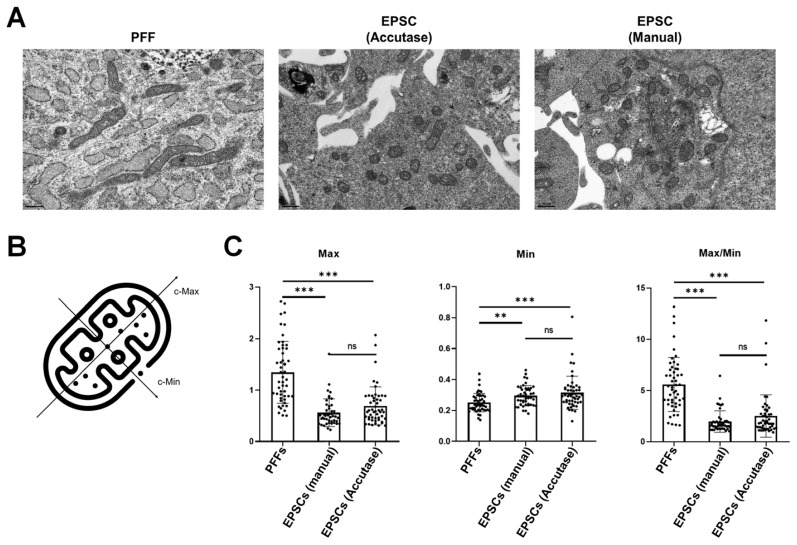
Analysis of mitochondrial morphology in PFFs and pEPSCs. (A) Transmission electron microscope (TEM) images of mitochondria in PFFs and pEPSCs with different sub-culture methods. Scale bars: 0.5 μm. (B) Illustration of criteria used for measuring mitochondrial axis length. (C) Analysis of the length of each axis of mitochondria in individual cells. Data are presented as mean±standard deviation for 25 independent mitochondrial samples. Sample sizes: PFFs, n = 51; pEPSCs (manual), n = 45; pEPSCs (Accutase), n = 50. PFFs, porcine fetal fibroblasts; EPSCs, expanded potential stem cells; pEPSCs, porcine expanded potential stem cells. ** p<0.002, *** p<0.001.

To compare the mitochondrial morphology in PFFs and pEPSCs, we conducted TEM analysis. As expected, PFFs exhibited elongated, rod-shaped mitochondria, whereas pEPSC displayed globular mitochondria, irrespective of the subculture protocol used (Figure 3A). To obtain precise measurements of mitochondrial morphology, we measured the “calculated-maximum (c-Max)” representing the longest length of mitochondria and “calculated-minimum (c-Min)” representing the shortest part of mitochondria (Figures 3B, 3C). PFFs had an average Max/Min value of 5.59, while pEPSCs subjected to Accutase and manual sub-culture showed values of 1.95 and 2.50, respectively. Max/Min value of PFFs is much higher than those of EPSCs (Figure 3D). However, there were no significant differences between the two subculture methods for EPSCs, indicating that the subculture method did not significantly affect mitochondrial morphology of EPSCs. Thus, we continued to use Accutase for single-cell passaging. These results indicate the successful remodeling of mitochondria from a rod-shaped to a globular-shaped morphology during the reprogramming of PFFs into pEPSCs.

### Comparison of mitochondrial metabolism profiles between porcine fetal fibroblasts and porcine expanded potential stem cells

**Figure 4:**
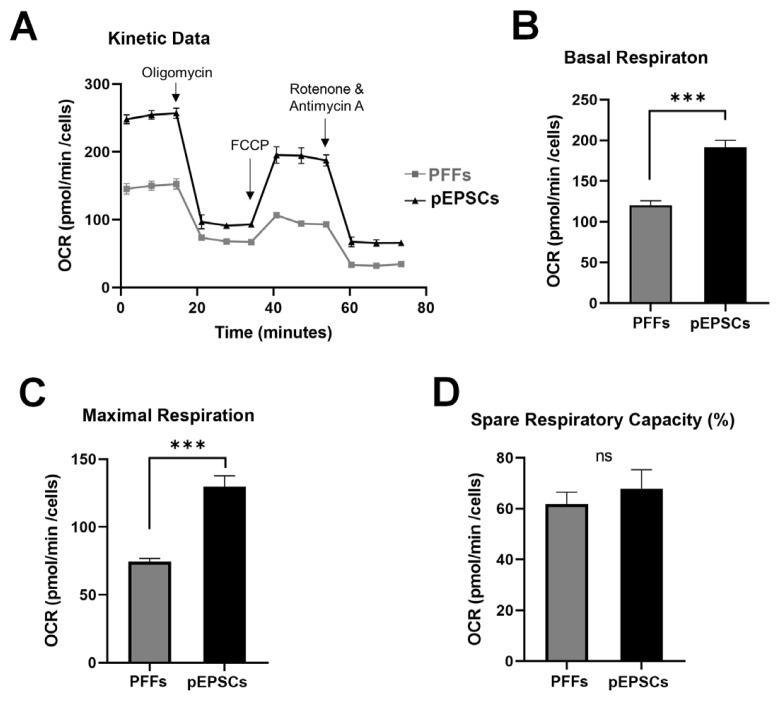
Mitochondrial respiratory function analysis in PFFs and pEPSC. (A) Measurement of oxygen consumption rate (OCR) in PFFs and pEPSC using Seahorse XFp analyzer. (B) Basal respiration (C) Maximal respiration (D) Spare Respiratory capacity (%). Data are presented as mean±standard deviation for n = 6 wells/group. PFFs, porcine fetal fibroblasts; pEPSCs, porcine expanded potential stem cells. Student’s t-test: ns = 0.298, *** p<0.001.

We analyzed the energy metabolism during the reprogramming of PFFs into pEPSCs using a Seahorse XFp analyzer. First, we assessed OXPHOS activity by measuring the OCR in both PFFs and pEPSCs (Figure 4A). Basal respiration, which is indicative of a cell’s energy demand under baseline conditions, was significantly higher in pEPSCs than in PFFs (Figure 4B). Following treatment with oligomycin (an ATP synthase inhibitor) and FCCP (a membrane potential decoupler), we measured maximal respiration and spare respiratory capacity (SRC) (Figures 4C, 4D). After FCCP treatment, both cell types showed increased OCR, with pEPSCs exhibiting significantly higher maximal respiration than PFFs. However, the SRC was not significantly different between the two cell types (Figure 4D), indicating that OXPHOS activity may not significantly differ between PFFs and pEPSCs.

**Figure 5:**
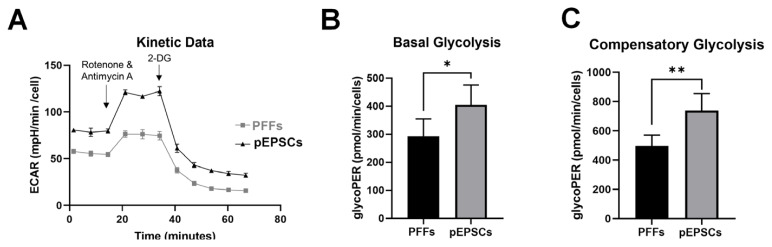
Glycolytic rate analysis (ECAR) in PFFs and pEPSC. (A) Measurement of extracellular acidification rate (ECAR) in PFFs and pEPSC. (B) Basal glycolysis (C) Compensatory glycolysis. Data are presented as mean±standard deviation for n = 6 wells/group. PFFs, porcine fetal fibroblasts; pEPSCs, porcine expanded potential stem cells. Student’s t-test: * p = 0.015, ** p = 0.001.

Next, we analyzed glycolytic activity using the ECAR (Figure 5A). Basal and compensatory glycolysis levels were significantly higher in pEPSCs than in PFFs (Figures 5B, 5C). Compensatory glycolysis was assessed by adding Rot/AA to inhibit respiration (OCR). These results collectively suggest that pEPSCs are more dependent on glycolysis than on OXPHOS compared to PFFs.

## DISCUSSION

In this study, PFFs were reprogrammed into pEPSCs using an integration-free method. The reprogrammed pEPSCs displayed key characteristics of EPSCs, including dome-shaped colony formation, expression of pluripotency markers, and the ability to differentiate into both embryonic and extraembryonic lineages. This confirms the successful reprogramming of PFFs. To assess differentiation capacity, pEPSCs underwent *in vitro* differentiation, which demonstrated their ability to form all three germ layers and extraembryonic lineages, underscoring their extended pluripotency. In addition, we investigated changes in mitochondrial morphology and energy metabolism during reprogramming. As anticipated, mitochondria in PFFs, which are typically elongated, transformed into a globular shape in pEPSCs. Energy metabolism analysis, involving the measurement of OCR for OXPHOS and ECAR for glycolysis, revealed that pEPSCs had significantly higher glycolysis levels compared to PFFs.

Conventional reprogramming methods primarily utilize viral vectors such as lentiviral or retroviral approaches, which integrate into the host genome. This integration leads to persistent genetic alterations and continuous transgene expression, which can significantly influence the transcriptome. Therefore, integration-free reprogramming methods are preferable for establishing high-quality iPSCs for potential clinical applications [22]. In this study, we reprogrammed PFFs using the Sendai virus system and further cultured them in LCDMV medium, supplemented with human leukemia inhibitory factor (hLIF), GSK3β inhibitor (CHIR99021), G protein-coupled receptors (GPCR) inhibitor (DiM), and PARP1 inhibitor (MiH) [7,21]. Increasing the concentration of CHIR99021 in LCDM culture medium successfully established EPSCs from porcine blastocysts [23]. This underscores the differences in the signaling pathways that maintain pEPSCs in humans and mice [23]. Therefore, analyzing molecular landmarks to optimize pEPSC culture media Although Nanog expression was minimal in ICC, RT-qPCR revealed approximately 20-fold higher *Nanog* expression in pEPSCs compared to PFFs, consistent with previous findings [7].

Reprogramming and differentiation involve dynamic changes in mitochondrial morphology and energy metabolism [20]. Therefore, we compared changes during the transition from PFFs to pEPSCs. Our data revealed that pEPSCs (preimplantation embryonic state) were more dependent on glycolysis than OXPHOS, compared to PFFs (differentiated somatic cells). As expected, the basal and compensatory glycolysis levels were much higher in pEPSCs than in PFFs. This observation aligns with the understanding that PSCs rely more on glycolysis than on OXPHOS for their energy metabolism [20,24].

Contrary to our expectation of lower OXPHOS activity in pEPSCs compared to PFFs, pEPSCs displayed higher basal and maximal respiration. Notably, the SRC of pEPSCs, which we anticipated to be higher, was similar to that of PFFs (Figure 4D). These findings are consistent with observations in mouse EPSCs and their neural stem cell derivatives, supporting the concept of a bivalent metabolic state in EPSCs [25]. Similar to naïve PSCs, EPSCs exhibit a bivalent state, using both glycolysis and OXPHOS for energy. The bivalent metabolic state of pEPSCs could be influenced by the components of the LCDMV culture medium. The Parp1 inhibitor MiH, involved in pathways related to pluripotency and reprogramming, plays a crucial role in maintaining EPSCs [5]. DiM, a GPCR inhibitor, affects histamine and muscarinic receptors [5,26]. The downstream effectors of these receptors are involved in MAPK signaling, which influences cell proliferation and differentiation [27]. Similarly, the GPCR antagonistic drug CM-20 protects mitochondria from oxidative stress and preserves mitochondrial function in human retinal pigment epithelium cells [28]. Therefore, MiH and DiM in the LCMDV medium might influence both the maintenance of pluripotency in pEPSCs by inhibiting ParP1 and GPCR and the energy metabolism of mitochondria. An alternative explanation for the similar OXPHOS activity could be the lower proliferation rate of the PFFs used for reprogramming. Their proliferation rate was half that of mesenchymal stem cells, potentially contributing to their lower reprogramming efficiencies (data not shown).

In conclusion, we successfully reprogrammed PFFs using an integration-free method and established pEPSCs that exhibited pluripotency gene expression and differentiation potential into both embryonic and extra-embryonic lineages, indicative of their expanded differentiation capabilities. Notably, we observed significant remodeling of mitochondrial morphology and a shift towards glycolytic energy dependence during the reprogramming of PFFs into pEPSCs. Further research is necessary to determine whether these pEPSC characteristics are conserved in other livestock. The established pEPSCs hold significant promise for various applications, including regenerative medicine, the development of genetically modified disease models, advancements in livestock breeding, and the production of cell-cultured meat.

## IMPLICATIONS

We demonstrate an integration-free method for establishing high-quality porcine stem cells, which holds promise for clinical applications and livestock improvement. Our findings reveal the extended pluripotency of pEPSCs and their capacity for differentiation into both embryonic and extraembryonic lineages, which is crucial for advancing regenerative medicine and basic research on embryonic and extraembryonic development in pigs — a challenge that has been difficult to address so far. Additionally, the established pEPSCs present opportunities for developing genetically modified disease models and advancing the production of cell-cultured meat, all of which are highly relevant to the field of animal science.

## Figures and Tables

**Table 1:** Antibodies used for immunocytochemistry

	List of antibodies used				

Type	Target protein	Origin	Manufacturer	Conjugate	Product
Primary	OCT4	Rabbit	Abcam		Ab19857
	SOX2	Rabbit	Millipore		AB5603
	NANOG	Rabbit	Abcam		Ab80892
	TUJ1	Mouse	Millipore		MAB1637
	BRACHYURY	Mouse	Santa Cruz		sc-166962
	SMA	Mouse	Abcam		Ab7817
	SOX17	Goat	R&D systems		AF1924
	GATA4	Rabbit	Abcam		Ab84593
	CDX2	Rabbit	Abcam		Ab76541
	EOMES	Rabbit	Abcam		Ab23345
	GATA3	Mouse	Santa Cruz		Sc-268
Secondary	Mouse IgG	Goat	Invitrogen	Alexa 488	A-11001
	Rabbit IgG	Goat	Invitrogen	Alexa 488	A-11008
	Goat IgG	Rabbit	Invitrogen	Alexa 568	A-11079
	Goat IgG	Donkey	Invitrogen	Alexa 647	A-21447

**Table 2:** Primer sets used for real-time qPCR

Genes	Primer sequence (5′-3′)	GeneBank ID	Size (bp)	Temperature (°C)
*pOCT4*	F GTGAGAGGCAACCTGGAGAG R GCGGACCACATCCTTCTCTA	NM_001113060.1	105	60
*pSOX2*	F CATCAACGGTACACTGCCTCTC R ACTCTCCTCCCATTTCCCTCTTT	NM_001123197.1	106	60
*pNANOG*	F TGAGGTTTATGGGCCTGAAG R CAGATCCATGGAGGAAGGAA	NM_001129971.2	103	60
*pGAPDH*	F TCCTGGGCTACACTGAGGAC R AGCTTGACGAAGTGGTCGTT	NM_001206359.1	112	60

qPCR, quantitative polymerase chain reaction; *OCT4*, octamer-binding transcription factor 4; *SOX2*, SRY-box transcription factor 2; *NANOG*, Nanog homeobox; *Gapdh*, glyceraldehyde-3-phosphate dehydrogenase.
